# *Streptomyces* sp. JCK-6131 Protects Plants Against Bacterial and Fungal Diseases via Two Mechanisms

**DOI:** 10.3389/fpls.2021.726266

**Published:** 2021-09-15

**Authors:** Khanh Duy Le, Jeun Kim, Hoa Thi Nguyen, Nan Hee Yu, Ae Ran Park, Chul Won Lee, Jin-Cheol Kim

**Affiliations:** ^1^Department of Agricultural Chemistry, Institute of Environmentally Friendly Agriculture, College of Agriculture and Life Sciences, Chonnam National University, Gwangju, South Korea; ^2^Department of Chemistry, Chonnam National University, Gwangju, South Korea

**Keywords:** *Streptomyces*, priming defense, streptothricin, ISR, SAR

## Abstract

Plant bacterial and fungal diseases cause significant agricultural losses and need to be controlled. Beneficial bacteria are promising candidates for controlling these diseases. In this study, *Streptomyces* sp. JCK-6131 exhibited broad-spectrum antagonistic activity against various phytopathogenic bacteria and fungi. *In vitro* assays showed that the fermentation filtrate of JCK-6131 inhibited the growth of bacteria and fungi with minimum concentration inhibitory (MIC) values of 0.31–10% and 0.31–1.25%, respectively. In the *in vivo* experiments, treatment with JCK-6131 effectively suppressed the development of apple fire blight, tomato bacterial wilt, and cucumber *Fusarium* wilt in a dose-dependent manner. RP-HPLC and ESI-MS/MS analyses indicated that JCK-6131 can produce several antimicrobial compounds, three of which were identified as streptothricin E acid, streptothricin D, and 12-carbamoyl streptothricin D. In addition, the disease control efficacy of the foliar application of JCK-6131 against tomato bacterial wilt was similar to that of the soil drench application, indicating that JCK-6131 could enhance defense resistance in plants. Molecular studies on tomato plants showed that JCK-6131 treatment induced the expression of the *pathogenesis-related* (*PR*) genes *PR1*, *PR3*, *PR5*, and *PR1*2, suggesting the simultaneous activation of the salicylate (SA) and jasmonate (JA) signaling pathways. The transcription levels of *PR* genes increased earlier and were higher in treated plants than in untreated plants following *Ralstonia solanacearum* infection. These results indicate that *Streptomyces* sp. JCK-6131 can effectively control various plant bacterial and fungal diseases via two distinct mechanisms of antibiosis and induced resistance.

## Highlights

We found two mechanisms of *Streptomyces* JCK-6131 effective protect the plant against bacterial and fungal diseases, including production of streptothricin-like antibiotics and priming in host defense.

## Introduction

Under field conditions, crops are often threatened by a variety of biotic pathogens including fungi, bacteria, nematodes, and viruses. Plant diseases caused by bacteria and fungi account for major losses in agricultural productivity (Lazarovits et al., 2014). Chemical bactericides and fungicides have been widely used to control various plant diseases. However, the misuse and indiscriminate use of chemical control agents have caused adverse effects on humans and the environment. Therefore, their application should be limited. Moreover, climate change and the rise of pesticide resistance also reduce the effectiveness of synthetic chemicals. This has led many scientists to search for potent alternative strategies against plant diseases (Ul Haq et al., 2020). The application of biological control methods is considered an effective alternative strategy (Lazarovits et al., 2014).

Plants have evolved a variety of defense mechanisms to protect themselves from pathogens, including physical and chemical defenses, which stop pathogen infection and the development of diseases (Nishad et al., 2020). Plants can alter their defense strategies depending on the pathogen. Plants distinguish and regulate their defense system based on the mechanism induced by the attacker and show suitable responses, such as the activation of the salicylic acid (SA) and jasmonic acid (JA)/ethylene (ET) pathways (De Vos et al., 2005). Plants interact with various microorganisms in the environment, and these interactions can be either positive or negative, either enhancing or compromising their defense system. Therefore, increasing research is being conducted on improving plant resistance against various pathogens (Bruce and Pickett, 2007).

Control of plant diseases using beneficial bacteria has great potential. Beneficial bacteria can enhance plant growth. Plant growth-promoting bacteria (PGPB) are classified according to their habitat; they can colonize between living tissues (endosphere), around the root (rhizosphere), on the surface of the root (rhizoplane), or on the aerial parts (phyllosphere) of plants (Mwajita et al., 2013; Dong et al., 2019). PGPB use various mechanisms to repress the growth of plant pathogens. They can directly repress pathogens by producing antibiotics, or through competition or parasitism. They can also indirectly repress pathogens by stimulating the plant’s defense system (Niu et al., 2011; Chaparro et al., 2014). For example, they can produce bioactive substances that can kill or inhibit the growth of pathogens. Till date, many studies have reported the production of natural chemicals, such as antibiotics, iron-chelating siderophores, antimicrobial volatiles, and lytic and detoxification enzymes, by beneficial bacteria (Compant et al., 2005). Additionally, some PGPB can stimulate defense resistance in plants against a variety of pathogens. Induction of defense resistance includes induced systemic resistance (ISR) and systemic acquired resistance (SAR), which can suppress plant diseases by up to 85% (Walters et al., 2013). It has been elucidated that the SA-dependent signaling pathway controls SAR through the induction of the expression of SA-responsive *pathogenesis-related* (*PR*) genes (e.g., *PR1*, *PR4*, and *PR5*), which are associated with broad-spectrum plant defense responses. In contrast, the JA/ET-dependent signaling pathway regulates ISR and is associated with the expression of JA/ET-responsive genes (e.g., *PR3*, *PR4*, and defensin [*PR12*]) (Van Loon and Van Strien, 1999). Treating plants with PGPB or their products (elicitors) enhances defense resistance against pathogens in the future; this process is known as priming (Conrath et al., 2015).

*Streptomyces* species are aerobic and filamentous bacteria able to produce a large number of antibiotics and bioactive compounds, some of which have been applied in controlling plant diseases (Dhanasekaran et al., 2012). Numerous studies have reported that *Streptomyces* spp. serve as PGPB and biocontrol agents in plant protection against bacterial and fungal diseases (Cheng et al., 2010; Suárez-Moreno et al., 2019; Vergnes et al., 2020). Moreover, *Streptomyces* spp. are capable of stimulating plant defense resistance (Dias et al., 2017; Abbasi et al., 2019; Vergnes et al., 2020). However, only a few *Streptomyces* strains have been studied thus far; this limits their potential use.

During screening for *Streptomyces* strains that show antagonistic activity against phytopathogenic bacteria and fungi, we discovered JCK-6131, a strain that exhibited a broad-spectrum antimicrobial activity, leading us to conduct this study. The main objectives of this research were to: (1) identify *Streptomyces* sp. JCK-6131, (2) examine the antimicrobial activity of its fermentation filtrate against phytopathogenic bacteria and fungi, (3) evaluate the bio-control efficacy of its fermentation broth against plant bacterial and fungal diseases, and (4) assess the induction of the expression of *PR* genes in tomato plants treated with the fermentation broth of *Streptomyces* sp. JCK-6131.

## Materials and Methods

### Culture and Fermentation of *Streptomyces*

*Streptomyces* sp. JCK-6131 was isolated from suppressive soil in Korea and maintained on international *Streptomyces* project 2 (ISP2; Becton, Dickinson, and Company, Franklin Lakes, NJ, United States) agar medium (yeast extract 4 g, malt extract 10 g, dextrose 4 g, agar 20 g, and distilled water for 1 L). The strain was stored in 30% glycerol at -80°C during the study period. For the purification of secondary metabolites, a single colony was transferred into ISP2 broth medium to make seed culture. After 48 h of incubation, 1% of the seed culture was inoculated into a 500-mL Erlenmeyer flask containing 100 mL of GSS medium (10 g soluble starch, 250 mg K_2_HPO_4_, 25 g soybean meal, 1 g beef extract, 4 g yeast extract, 2 g NaCl, 20 g glucose, 2 g CaCO_3_, and distilled water for 1 L of medium, pH adjusted to 7.2) and fermented at 30°C and 180 rpm for 3 days. The obtained fermentation broth was centrifuged to divide the pellet and supernatant for further experiment.

### Plant Pathogenic Microorganisms

Sixteen phytopathogenic bacteria were used in this study, including *Acidovorax avenae* subsp. *cattleyae* (Aac), *Acidovorax konjaci* (Ak), *Agrobacterium tumefaciens* (At), *Burkholderia glumae* (Bg), *Clavibacter michiganensis* subsp. *michiganensis* (Cmm), *Erwinia pyrifoliae* (Ep), *Erwinia amylovora* (Ea), *Pectobacterium carotovorum* subsp. *carotovorum* (Pcc), *Pectobacterium chrysanthemi* (Pc), *Pseudomonas syringae* pv. *lachrymans* (Psl), *Pseudomonas syringae* pv. *actinidiae* (Psa), *Ralstonia solanacearum* (Rs), *Xanthomonas euvesicatoria* (Xe), *Xanthomonas axonopodis* pv. *citri* (Xac), *Xanthomonas arboricola* pv. *pruni* (Xap), and *Xanthomonas oryzae* pv. *oryzae* (Xoo). The bacteria were kindly supplied by Dr. S. D Lee of Rural Development Administration, Prof. S.-W. Lee of Dong-A University, and Prof. Y-G Ko of Suncheon National University, South Korea and stored in 30% glycerol at -80°C in Chonnam National University. The bacteria were cultured on tryptic soy agar (TSA; Becton, Dickinson, and Company) and tryptic soy broth (TSB; Becton, Dickinson, and Company) media at 30 ± 2°C. Four phytopathogenic fungi—*Botrytis cinerea* (Bc), *Colletotrichum coccodes* (Cc), *Fusarium oxysporum* f. sp. *cucumerinum* (Foc), and *Rhizoctonia solani* (Rhs)—were obtained from the Korea Research Institute of Chemical Technology, Daejeon, South Korea. *Raffaelea quercus-mongolicae* (Rq) and *Sclerotinia homoeocarpa* (Sh) were kindly supplied by the Korea Forest Research Institute, Seoul, South Korea. *Fusarium graminearum* (Fg) and *Pythium ultimum* (Pyu) were obtained from Seoul National University, Seoul, and Korea Agricultural Culture Collection, Jeonju, South Korea, respectively. All fungi were incubated on potato dextrose agar (PDA; Becton, Dickinson, and Company) and potato dextrose broth (PDB; Becton, Dickinson, and Company) media at 25°C.

### Plant Materials

Tomato (cultivar “Seokwang”; Farmhannong Co., Ltd, Seoul, South Korea) was sown in vinyl pots (6.0 cm diameter) containing nursery soil and kept in an incubation room with 12 h daylight per day for 5 weeks. Cucumber (cultivar “Chungboksamchok”; Syngenta, South Korea) was soaked in warm water for 2 h then germinated in a petri dish containing wet paper at 30°C for 2 days. The germinated seeds were sown into nursery soil and grown at 25°C under the same light conditions for 7 days. *Arabidopsis thaliana* (*PR1:GUS* transgenic) seeds were surface-sterilized in a solution of 25% bleach plus 0.01% tween 20 for 5 min, followed by 70% ethanol for 5 min. Then, seeds were rinsed five times and soaked in sterilized water at 4°C for 2 days. These seeds were germinated on Murashige-Skoog (MS) agar medium (0.43 g MS salt, 1 g sucrose, and 0.7 g phyto-agar per 100 mL, pH 5.7) plus 50 μg/mL of kanamycin for 2 weeks at 25°C with a cycle of 16 h daylight per day in a growth-chamber.

### Sequence and Whole-Genome Comparative Analysis

For identification of JCK-6131 strain, total DNA of JCK-6131 was extracted using an I-genomic BYF DNA Extraction mini kit (iNtRON Biotechnology, Inc., Seongnam-si, Gyeonggi-do, South Korea). The 16S rRNA was amplified by PCR using the primer pair 27f 5′- AGAGTTTGATCCTGGCTCAG – 3′ and 1492r 3′ – TTCAGCATTGTTCCATTGG – 5′. The PCR thermal profile was 95°C for 5 min; 30 cycles of 95°C for 30 s, annealing at 55°C for 30 s, and 72°C for 1 min 30 s; and a final extension at 72°C for 10 min. The sequence (1385 bp) was compared for similarity with the reference species of *Streptomyces* in the GenBank database using the BLAST search tool. Phylogenetic analysis was performed using the neighbor-joining DNA distance algorithm with MEGA (version X). Additionally, the whole gene sequences were uploaded and pairwise genome calculations of OrthoANI were performed using the Orthologous Average Nucleotide Identity tool (Lee et al., 2016).

### Biochemical and Physiological Identification

The strain JCK-6131 was cultured on ISP media (ISP1, 2, 3, 4, 5, and 7) to observe the color of the colony and pigment production. The ability to enzymatic production, utilization of multiple carbon and nitrogen sources were also evaluated. The physiological tests were performed to estimate the growth of JCK-6131 on different concentrations of NaCl, temperatures as well as pH (Lima et al., 2017).

### *In vitro* Antimicrobial Assay

The antimicrobial activity of the fermentation filtrate of *Streptomyces* sp. JCK-6131 was examined against all test microorganisms using a broth dilution method according to previous reports for bacteria (Le et al., 2020) and fungi (Kim et al., 2020). A serial two-fold dilution of the fermentation filtrate (10, 5, 2.5, 1.25, 0.63, 0.31, 0.16, and 0.08%) was prepared for these assays. Streptomycin sulfate was used as a positive control for phytopathogenic bacteria. The test microorganisms were incubated at 30°C for 1–2 days for bacteria and 25°C for 3–7 days for fungi. The minimum inhibitory concentration (MIC) value was the lowest concentration that can inhibit the growth of microorganisms. Each run of the experiment contained three replicates, and the entire experiment was repeated twice.

### Isolation and Identification of Antimicrobial Metabolites

The fermentation broth was centrifuged to remove the cell mass and then the pH of the supernatant (1 L) was adjusted to 3.6 using 5 M HCl. Amberlite XAD-16N resin (50 g) was added to the culture supernatant and then the suspension was kept overnight, followed by filtering through a filter paper. The filtrate was precipitated in ice acetone (1:4, v/v), and the precipitate was obtained by centrifugation. The precipitate was dissolved in PBS (50 mM, pH 7.0) then loaded on a Sephadex LH20 open column that equilibrated and eluted the sample using water. Fractions that showed antibacterial activity against indicator bacteria were combined to get partially purified extract (PPE), and PPE were further purified by reverse-phase high-performance liquid chromatography (RP-HPLC; Shimadzu, Japan). The purified substances were collected and lyophilized based on their antibacterial activity, and their molecular masses were analyzed by liquid chromatography-mass spectrometry (LC-MS) (Shimadzu 6AD HPLC; API2000) in positive mode (100–1800 *m/z*) with a ZORBAX C18 column (4.6 × 250 mm). The mobile phases were distilled water and ACN containing 0.05% TFA. A linear gradient of 0–8% over 16 min was used with a flow rate of 1 mL/min. The highly purified samples were used for electrospray ionization tandem mass spectrometry (ESI-MS/MS). The mass parameters were as follows: curtain gas, 30; spray voltage, 5500; ion source gas, 50 psi; and flow rate, 10 μL/mL.

### Historical GUS Staining Assay

*Arabidopsis* seedlings were transplanted to a liquid medium containing either fermentation broth or fermentation filtrate of *Streptomyces* sp. JCK-6131 at a final concentration of 1%. The plants were placed in a growth chamber with 12 h daylight per day at 25°C for 2 days. Salicylic acid (SA) was used as a positive control and GSS medium as a negative control. Historical GUS staining was conducted as previously reported (Kondo et al., 2014). Briefly, the seedlings were fixed in 90% ice acetone at -20°C for 1 h and washed twice in sodium phosphate buffer (pH 7.0). Washed plants were immersed in GUS staining solution that consisted of 0.1% Triton-X, 100 mM sodium phosphate buffer, 2.5 mM potassium ferrocyanide, 2.5 mM potassium ferricyanide, and 2 mM X-GlucA (Duchefa, X1405) overnight at 30°C. Reactions were stopped by 70% ethanol for 30 min and the stained samples were washed in 90% ethanol several times to remove chlorophyll. The GUS activity was qualitatively assayed by determining the blue color and observed under an optical microscope.

### RNA Isolation and RT-qPCR

Tomato plants were sprayed with the 100-fold-diluted fermentation broth of *Streptomyces* sp. JCK-6131 at 3 days before Rs inoculation. Three plants from each group were taken at 1, 2, and 3 days after inoculation. The leaves were collected and ground in liquid nitrogen using a porcelain mortar. Total RNA was extracted using an Iqeasy^TM^ Plant RNA Extraction mini kit (iNtRON, Korea) and quantified using a NanoDrop spectrometer (NP80, IMPLEN, Germany). cDNA was synthesized from 1 μg of each RNA sample after treating with RNase-free DNase I using a SuperScript^TM^ IV First-Strand Synthesis System kit (Invitrogen). RT-qPCR was performed using a total reaction volume of 10 μL, containing 1 μL of cDNA template, 4 μL of 0.625 pM of forward and reverse primers, and 5 μL of 2 × iQ^TM^ SYBR Green supermix (BioRad) on a BioRad CFX96^TM^ Real-Time System. The PCR cycling parameters were denaturation at 95°C for 3 min; followed by 40 cycles at 95°C for 9 s, 55°C for 1 min, and 55°C for 3 s; and a final extension at 95°C for 30 s. Data was expressed as the normalized ratio of target genes to the internal reference *Ubiquitin* (*UBI*) gene. Primer sequences are provided in Supplementary Table 1. Experiments were conducted in triplicate.

### *In vivo* Antimicrobial Assays

#### Preparation of Samples

The biocontrol efficacy of the fermentation broth of *Streptomyces* sp. JCK-6131 was evaluated against tomato bacterial wilt, fire blight of apple, and *Fusarium* wilt of cucumber. The fermentation broth was diluted in distilled water at 5-, 10-, 20-, and 100-fold, and then, Tween 20 was incorporated into each diluent at a concentration of 250 μg/mL. Additionally, PPE was also prepared in Tween 20 solution at 1,000, 100 and 10 μg/mL. Buramycin (streptomycin sulfate 20% WP; Farm Hannong Co., Seoul, Korea) and janroken (hymexazol 30% + penthiopyrad 5% WP; Hankook Samgong Co., Seoul, Korea) were used as positive controls for bacterial and fungal diseases, respectively. Tween 20 solution was used as a negative control.

#### Bacterial Wilt Disease of Tomato

During the fourth-leaf stage of tomato plants with bacterial wilt, soil was drenched with the fermentation broth of *Streptomyces* sp. JCK-6131 and PPE. The plants were treated with 20 mL of each sample per pot at 24 h before inoculation, and then 20 mL of Rs cell suspension (10^8^ CFU/mL, 10 mM MgCl_2_) was inoculated by soil drenching. A separate experiment was designed to evaluate the potential of ISR in tomato by *Streptomyces* sp. JCK-6131 against Rs infection. The tomato plant leaves were treated with the 100-fold-diluted fermentation broth and the fermentation filtrate three days before inoculation by foliar spraying. The inoculated plants were kept in the dark for 24 h and then transferred to an incubation room at 30°C and 75% RH with 12 h daylight per day for 10 days. The disease index was graded on the following scale: 0 = no leaf symptom, 1 = one leaf wilted, 2 = two or three leaves wilted, 3 = more than four leaves wilted, and 4 = plant dead (Le et al., 2020).

#### Fire Blight Disease of Apple

The biological efficacy of *Streptomyces* sp. JCK-6131 was evaluated against fire blight disease of apple using a detached leaf assay. The youngest true leaves were excised from the tips of growing shoots of 3-year-old apple trees (Hongro cultivar, moderately sensitive to fire blight disease) and sterilized in 1% bleach solution for 1 min, followed by rinsing three times in sterile water. The leaves were air-dried in a clean-beach, and then the detached leaf assay of fire blight disease was performed according to the method of Cabrefiga and Montesinos (2017). Briefly, each leaf was wounded with a sterile knife (or forceps) in the lamina in four positions, followed by treating with 10 μL of each sample onto each wound. After 1 h of air-drying, 10 μL of Ea suspension (10^7^ CFU/ml) was inoculated at each pretreated position. The disease severity was recorded on the following scale: 0, no symptom; 1, necrosis located around the wound; 2, necrosis progressing far from the wound; and 3, necrosis of the whole leaf.

#### *Fusarium* Wilt Disease of Cucumber

The assay was conducted on seven-day-old seedlings of cucumber according to the method of Lee et al. (2014). The seedlings were pretreated with 20 mL of each sample per pot by soil drenching, and then the treated plants were inoculated with 20 mL solution of conidial suspension of Foc (10^6^ conidia/ml) 24 h after treatment by soil drenching. The inoculated plants were kept in darkness for 24 h and incubated at 25°C and 75% RH with 12 h daylight per day for 4 weeks in an incubation room. The severity was calculated using the following scale: 0, no symptoms; 1, < 25% of leaves showing yellowing and or necrosis; 2, 26–50% of leaves showing yellowing and/or necrosis; 3, 51–75% of leaves showing yellowing and/or necrosis; and 4, 76–100% of leaves showing wilt, yellowing, and/or necrosis (Abro et al., 2019).

#### Calculation of Control Value

The *in vivo* experiments were repeated two times with three replicates per treatment and disease severity (DS) was transferred to a control percentage (±standard deviation) compared to the untreated control using the following equation:


(1) $$\mathrm{Control}\mathrm{value}\left(\%\right)=\frac{\left(\mathrm{DS}\mathrm{of}\mathrm{untreated}\mathrm{control}-\mathrm{DS}\mathrm{of}\mathrm{treatment}\right)}{\mathrm{DS}\mathrm{of}\mathrm{untreated}\mathrm{control}}\times 100\%.$$

### Statistical Analysis

Results of the pot experiments were analyzed by one-way analysis of variance (ANOVA), and significant differences among treatments were evaluated with Fisher’s least significant difference test. The comparison of two treatment methods was conducted by Student *t*-tests. *In vitro* data were analyzed by ANOVA with Duncan’s multiple range test. A *p*-value of < 0.05 was considered significant. The statistical analysis was performed in SPSS 23.0 software (SPSS Inc. Chicago, IL, United States).

## Results

### Taxonomical Identification of *Streptomyces* sp. JCK-6131

**FIGURE 1:**
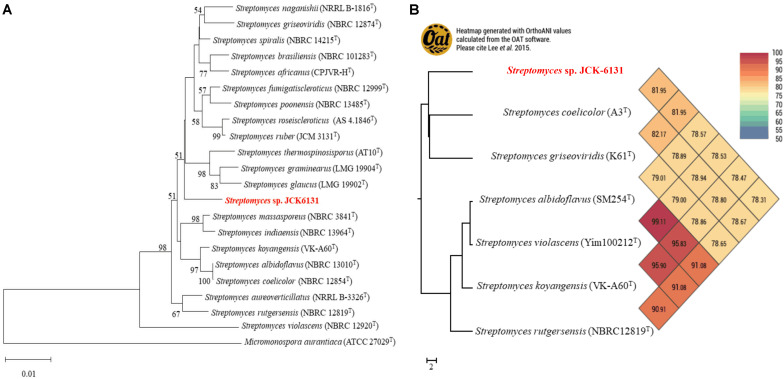
Taxonomic classification of genome comparative of *Streptomyces* sp. JCK6131 compared to the genome of other related *Streptomyces.* **(A)** Phylogenetic relationships between JCK-6131 and related *Streptomyces* strains represented as a neighbor-joining (NJ) tree based on 16S rRNA gene sequences. The evolution history was determined using the NJ method with a Jukes-Cantor model. The numbers at the branching points represent the bootstrap values from 1,000 replications. *Micromonospora aurantiaca* ATCC 27029^*T*^ was used as the outgroup. Bar = 0.010 substitutions per nucleotide position. **(B)** Heatmap generated using the OrthoANI values for JCK-6131 and other type strains of *Streptomyces* species calculated using the OAT software.

The partial 16S rRNA (1385-bp) gene sequence of JCK-6131 was deposited in GenBank under accession number MW911616. Comparison of 16S rRNA sequences indicated that JCK-6131 was closely related to *Streptomyces spiralis* NBRC 14215 and *Streptomyces thermospinosisporus* AT10, with similarity values of 98.34 and 98.13%, respectively. However, the phylogenetic tree analysis based on neighbor-joining algorithms indicated that JCK-6131 could not be identified as a known *Streptomyces* species (Figure 1A). Also, the ANI calculation was determined using complete genomes of six *Streptomyces* strains available in NCBI which included *Streptomyces coelicolor* A3^*T*^, *Streptomyces griseoviridis* K61^*T*^, *Streptomyces albidoflavus* SM254^*T*^, *Streptomyces violascens* Yim1000212^*T*^, *Streptomyces koyangensis* VK-A60^*T*^, and *Streptomyces rutgersensis* NBRC12819^*T*^ (Figure 1B). Low relative identity values were obtained when comparing the genomic similarity between JCK-6131 and other type strains of the genus *Streptomyces*. OrthoANI values ranged from 78.31 to 81.95% (<95%), representing a different genome-species with other closely related *Streptomyces* strains within classified groups. The results clearly proved that *Streptomyces* sp. JCK-6131 represents a novel species in the genus *Streptomyces*. Additionally, the biochemical and physiological tests showed that the JCK-6131 have the ability to utilize multiple carbon and nitrogen sources, and growth on a wide range of temperature (15–45^*o*^C, opt 30^*o*^C), pH (5–10, opt 7), and concentration of NaCl up to 6%. Together with that, the JCK-6131 strain could produce some hydrolysis enzyme such as protease, lipase, amylase, and cellulase. Further studies for species identification showed that color of colony and pigment production of JCK-6131 on ISP media that also were different from *Streptomyces spiralis* (Supplementary Table 2).

### Antagonistic Activity of JCK-6131 Against Plant Pathogenic Bacteria and Fungi

*Streptomyces* sp. JCK-6131 exhibited broad-spectrum antibacterial activity against all test pathogenic bacteria. Among the 16 test bacteria, Aac and Ak were the most sensitive to the fermentation filtrate with an MIC value of 0.31%. Cell growth of Ea, Ep, Xoo, and Rs were highly inhibited, with an MIC value of 1.25%, followed by Pcc, Psa, Xe, Xap, and Xac, which had an MIC value of 2.5%. Moderate sensitivity was recorded against Psl and Pc, with an MIC range of 3.33–4.17%. Three bacterial strains—At, Bg, and Cmm—were relatively insensitive to the fermentation filtrate (Table 1).

**TABLE 1:** Minimum inhibitory concentration (MIC) of the fermentation filtrate of *Streptomyces* sp. JCK-6131 against several plant pathogenic bacteria.

Plant pathogenic bacteria	MIC	
	Fermentation filtrate (%)	Streptomycin sulfate (μ g/mL)
*Acidovorax avenae* subsp. *cattleyae*	0.31 ± 0.00^e^	500 ± 0.00^b^
*Acidovorax konjaci*	0.42 ± 0.18^e^	7.81 ± 0.00^e^
*Agrobacterium tumefaciens*	10.00 ± 0.00^a^	166.67 ± 72.17^c^
*Burkholderia glumae*	10.00 ± 0.00^a^	20.84 ± 9.02^e^
*Clavibacter michiganensis* subsp. *michiganensis*	10.00 ± 0.00^a^	20.84 ± 9.02^e^
*Erwinia amylovora* TS3128	2.50 ± 0.00^bcd^	5.21 ± 2.25^e^
*Erwinia pyrifoliae*	3.33 ± 1.44^bc^	5.21 ± 2.25^e^
*Pectobacterium carotovorum* subsp. *carotovorum*	3.33 ± 1.44^bc^	15.63 ± 0.00^a^
*Pectobacterium chrysanthemi*	4.17 ± 1.44^b^	500 ± 0.00^b^
*Pseudomonas syringae* pv. *actinidiae*	3.33 ± 1.44^bc^	15.63 ± 0.00^e^
*Pseudomonas syringae* pv. *lachrymans*	3.33 ± 1.44^bc^	15.63 ± 0.00^e^
*Ralstonia solanacearum*	1.25 ± 0.00^de^	10.42 ± 4.52^e^
*Xanthomonas euvesicatoria*	2.50 ± 0.00^bcd^	20.83 ± 9.02^e^
*Xanthomonas arboricola* pv. *pruni*	2.50 ± 0.00^bcd^	62.5 ± 0.00^d^
*Xanthomonas campestris* pv. *citri*	3.33 ± 1.44^bc^	31.25 ± 0.00^e^
*Xanthomonas oryzae* pv. *oryzae*	1.25 ± 0.00^de^	10.42 ± 4.52^e^

*Means within the same column followed by a different letter are significantly different (*p* < 0.05), according to Duncan’s multiple range test. The names of bacteria are presented in alphabetical order.*

In addition, the fermentation filtrate of *Streptomyces* sp. JCK-6131 also inhibited the mycelial growth of eight phytopathogenic fungi. Bc was the most sensitive, followed by Fg and Rs. The mycelial growth of Rq, Foc, and Sh was moderately inhibited by the fermentation filtrate. Cc was the most insensitive to the fermentation filtrate (Table 2).

**TABLE 2:** Minimum inhibitory concentration (MIC) of the fermentation filtrate of *Streptomyces* sp. JCK-6131 against eight plant pathogenic fungi.

Plant pathogenic fungi	Fermentation filtrate (%)
*Botrytis cinerea*	0.42 ± 0.18^d^
*Colletotrichum coccodes*	1.67 ± 0.72^a^
*Fusarium graminearum*	0.63 ± 0.00^cd^
*Fusarium oxysporum* f. sp. *cucumerinum*	1.25 ± 0.00^ab^
*Pythium ultimum*	1.04 ± 0.36^bc^
*Raffaelea quercus-mongolicae*	1.04 ± 0.63^bc^
*Rhizoctonia solani*	0.63 ± 0.00^cd^
*Sclerotinia homoeocarpa*	1.25 ± 0.00^ab^

*Means within the same column followed by a different letter are significantly different (*p* < 0.05), according to Duncan’s multiple range test. The names of the fungi are presented in alphabetical order.*

### Identification of Antimicrobial Metabolites

**FIGURE 2:**
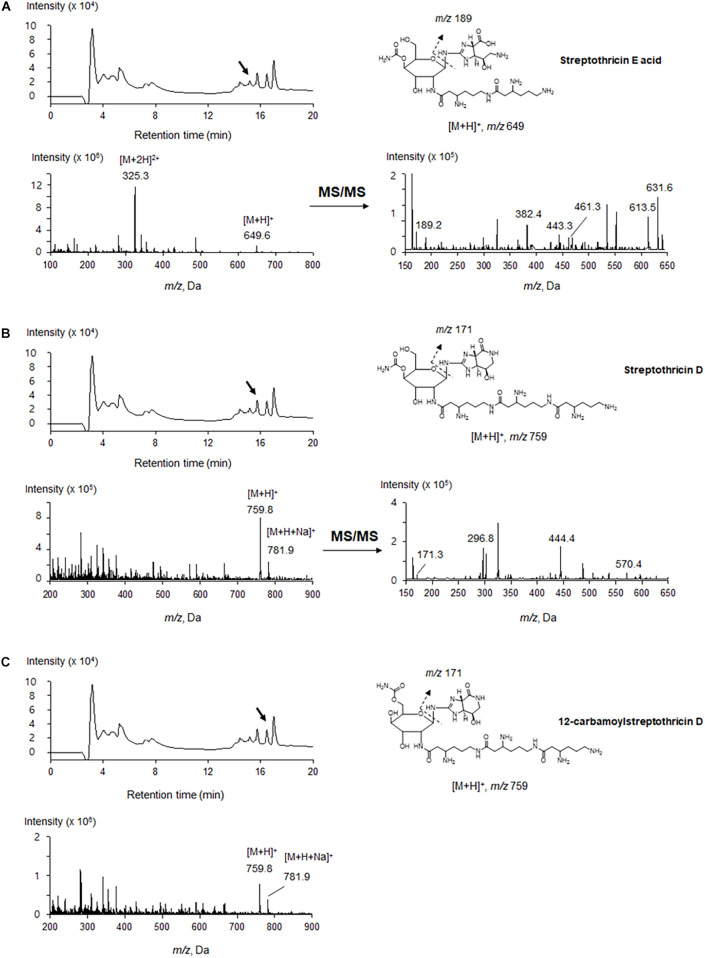
LC-ESI-MS analysis of the compounds produced by *Streptomyces* sp. JCK-6131. Streptothricin E acid **(A)**, streptothricin D **(B)**, and 12-carbamoylstreptothricin D **(C)**. The LC chromatograms, mass spectra, and chemical structures with fragmentation patterns are shown.

The antimicrobial compounds remained in the aqueous layer after being partitioned with organic solvents, and they positively reacted with ninhydrin, suggesting that JCK-6131 produced amino acid-containing antimicrobial compounds. According to the mass analysis, we found that the partially purified fraction contained at least three compounds, including [M + H]^+^ ion peaks at 649.6 (Figure 2A) and 759.8 *m/z* (Figures 2B,C). Each compound was predicted to correspond to streptothricin E acid, streptothricin D, and 12-carbamoylstreptothricin D, respectively, based on the collision-induced dissociation (CID) spectra of each compound by ESI-MS/MS analysis (Figure 2; Ji et al., 2008, 2009). Streptothricin E acid presented multiple product ions at 189.2 [S′], 382.4 [GLL-2H_2_O-NH_2_CO], 443.3 [GLL-H_2_O], 461.3 [GLL], 613.5 [M-2NH_3_], and 631.6 *m/z* [M-NH_3_] (Supplementary Figure 1). Streptothricin D and 12-carbamoylstreptothricin D presented the same fragment ions at 171.3 [S], 296.8 [GS-2H_2_O-NH_2_CO], 444.4 [GLL-H_2_O], and 570 *m/z* [GLLL-H_2_O] (Supplementary Figures 2, 3). The structures of streptothricin consist of a streptolidine (S/S’), a carbamoylated D-gulosamine (G), and a β-lysine chain (L) Ji et al. (2008).

### Efficacy of *Streptomyces* sp. JCK-6131 in Controlling Plant Bacterial and Fungal Diseases

**FIGURE 3:**
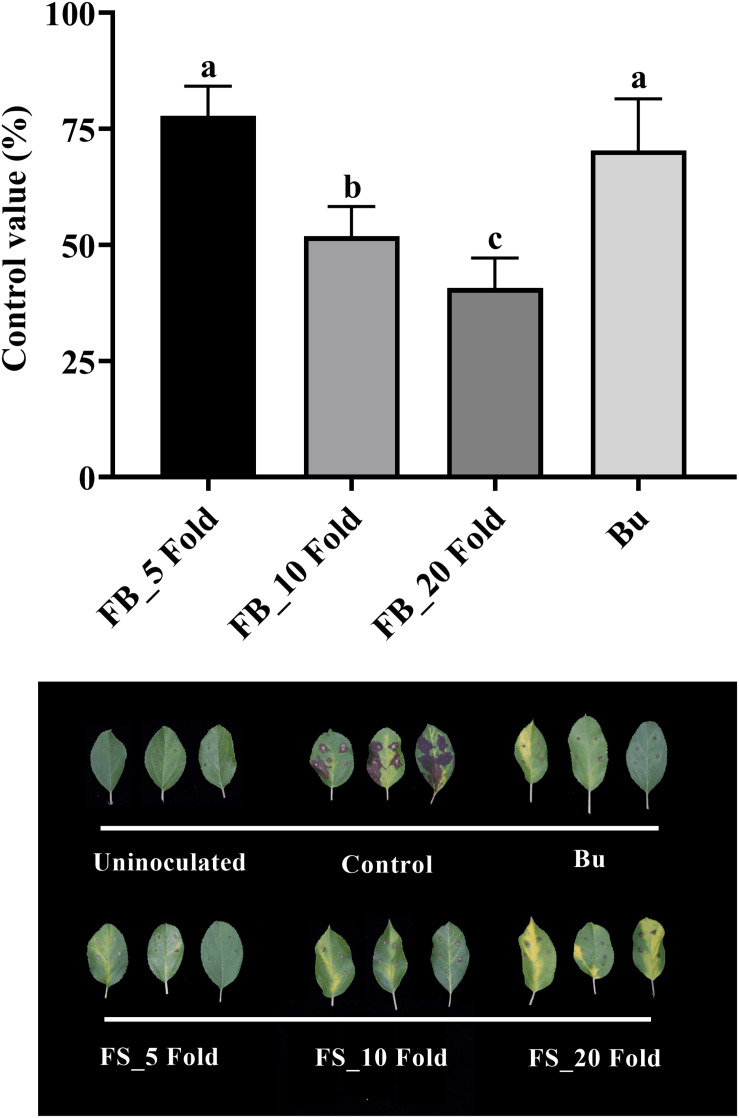
Control efficacy of the fermentation broth of *Streptomyces* sp. JCK-6131 against apple fire blight in a detached leaf assay. FB = fermentation broth of *Streptomyces* sp. JCK-6131 and Bu = buramycin (×1000). Values are presented as the mean ± standard error of three runs, with three replicates each. The bars with the same letters represent non-significant differences between the treatments (*p* < 0.05, Fisher’s least significant difference test).

**FIGURE 4:**
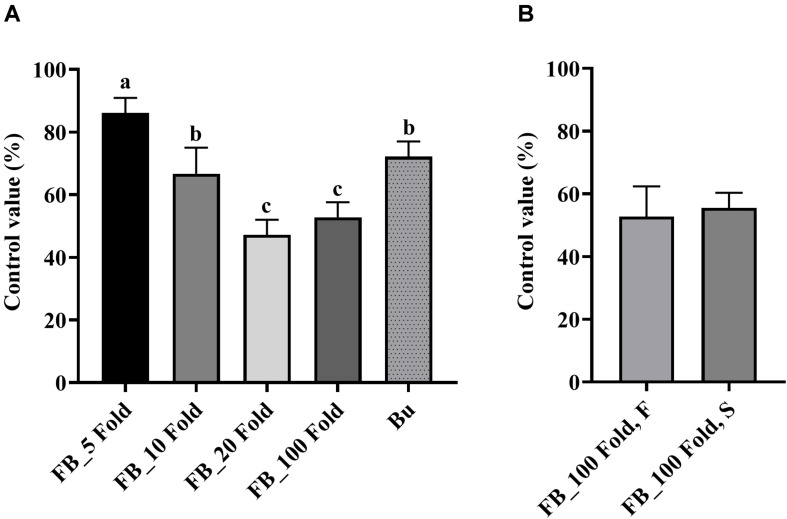
Control efficacy of the fermentation broth of *Streptomyces* sp. JCK-6131 against tomato bacterial wilt in a pot experiment. **(A)** Treatment by soil drenching 1 day before inoculation. FB = fermentation broth of *Streptomyces* sp. JCK-6131, Bu = buramycin (×1000); analyzed by Fisher’s least significant difference test. **(B)** Treatment by foliar spraying (F) and soil drenching (S) 3 days before inoculation; analyzed by student *t*-test. Values are presented as the mean ± standard error of three runs, with three replicates each. The bars with the same letters represent non-significant differences between the treatments (*p* < 0.05).

Results of the detached leaf assay showed that the fermentation broth suppressed the development of apple fire blight caused by Ea in a dose-dependent manner (Figure 3). The highest protective activity was obtained by the treatment with the 5-fold-diluted fermentation broth of *Streptomyces* sp. JCK-6131 with a control value of 77.78% (*F*_3,8_ = 13.94, *p* < 0.01), which was not significantly different from the positive control buramycin treatment (*p* = 0.549). Likewise, the treatment with the 5-fold-diluted fermentation broth significantly reduced the severity of tomato bacterial wilt caused by Rs, with a control value of 86.11% (*F*_4,10_ = 13.32, *p* < 0.001), which was significantly higher than that of buramycin (*p* = 0.014) (Figure 4A). Interestingly, there was no significant difference in disease control between the treatment with the 20-fold-diluted fermentation broth and that with the 100-fold-diluted fermentation broth (*p* = 0.260), indicating the possibility of disease control efficacy via the ISR-activating effects of the fermentation broth of *Streptomyces* sp. JCK-6131. Hence, we further conducted the *in vivo* bioassay against tomato bacterial wilt, where the 100-fold-diluted fermentation broth was applied to the test plants by either soil drenching or foliar spraying. Both treatments suppressed the development of disease symptoms to similar extents, with a disease control efficacy of 55.55% for soil drenching and 52.78% for foliar spraying, which were not significantly different (*p* = 0.678) (Figure 4B).

On the other hand, PPE also reduced the development of tomato bacterial wilt disease in a dose-dependent manner with the highest control value of 88.89% by the treatment at 1,000 μg/mL (*F*_3,8_ = 5.11, *p* < 0.05, Supplementary Figure 4). Even at a low concentration of 10 μg/mL, it could control the plant disease with a control value of 58.33%.

**FIGURE 5:**
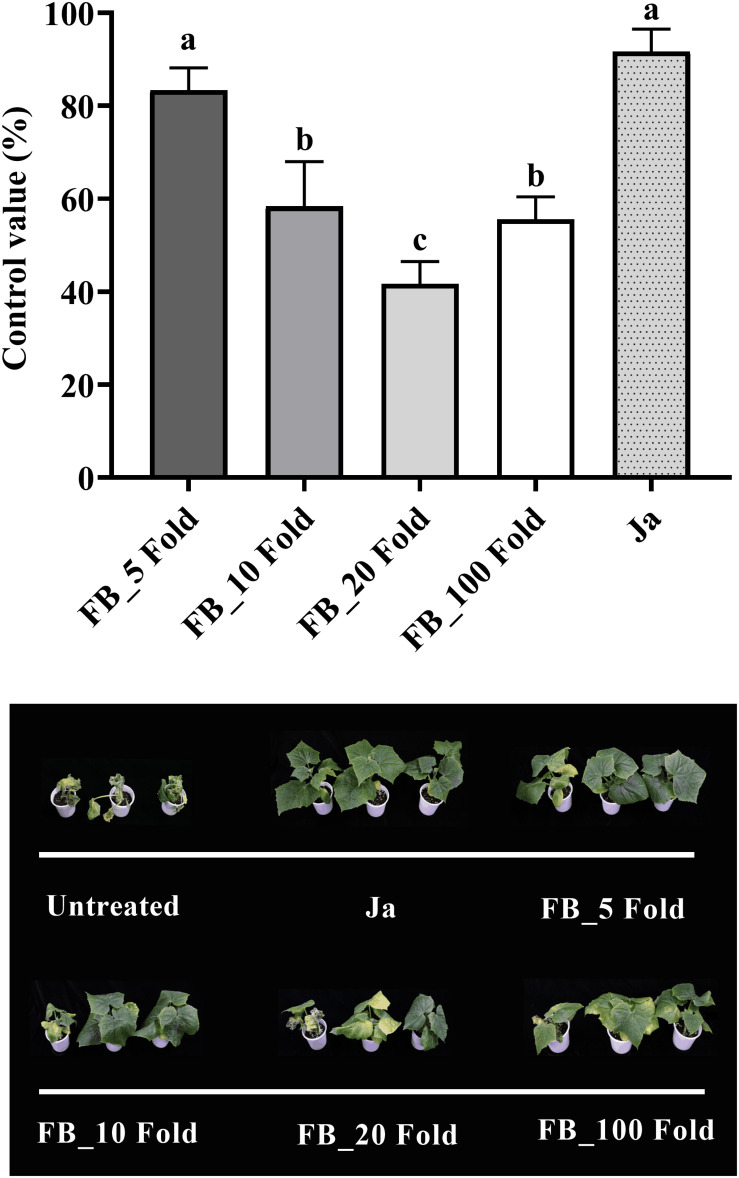
Control efficacy of the fermentation broth of *Streptomyces* sp. JCK-6131 against cucumber *Fusarium* wilt in a pot experiment. FB = fermentation broth of *Streptomyces* sp. JCK-6131, Ja = janroken (×1000). Values are presented as the mean ± standard error of three runs, with three replicates each. The bars with the same letters represent non-significant differences between the treatments (*p* < 0.05, Fisher’s least significant difference test).

The fermentation broth of *Streptomyces* sp. JCK-6131 also effectively suppressed the development of cucumber *Fusarium* wilt disease. The control efficacy was positively correlated with the concentration of fermentation broth at all dilutions between 5- and 20-fold, but not at 100-fold dilution (Figure 5). The development of *Fusarium* wilt on cucumber was significantly repressed by the treatment with the 5-fold-diluted fermentation broth, with a control value of 83.33% (*F*_4,10_ = 23.67, *p* < 0.001), which was quite similar to that of the positive control janroken treatment (*p* = 0.125). The treatment with the 100-fold-diluted fermentation broth also displayed a control value similar to that of the treatment with the 10-fold-diluted fermentation broth (*p* = 0.588).

### Qualitative Induction of PR1 Gene Expression

**FIGURE 6:**
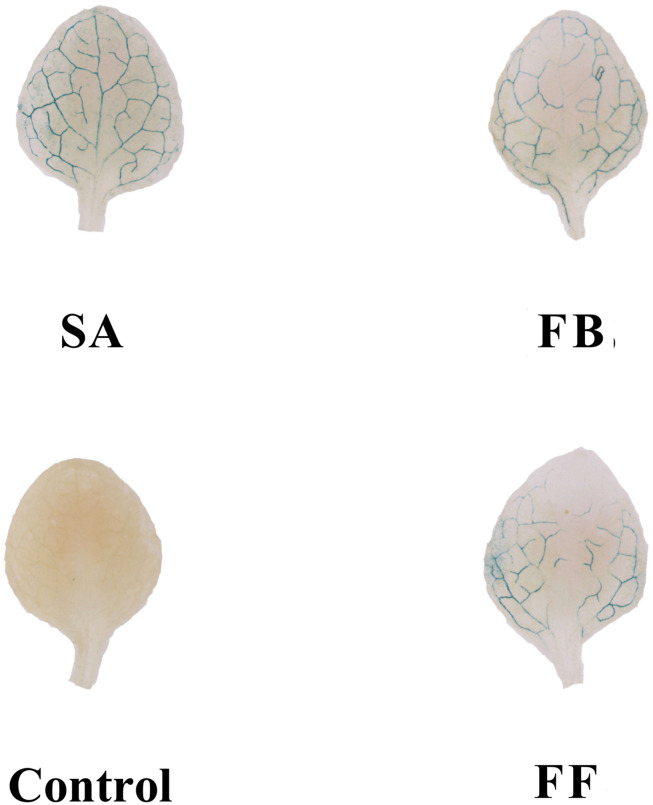
GUS staining assay of *Arabidopsis* transgenic (PR1:GUS) seedlings treated with the fermentation broth (FB) and fermentation filtrate (FF) of *Streptomyces* sp. JCK-6131. SA = salicylic acid (0.1 mM), 1% GSS medium was used as a negative control.

Based on GUS activity analysis, either fermentation broth or filtrate treatment resulted in induction of *PR1:GUS* gene expression, which was slightly weaker than that of the SA control. In contrast, no expression of the *PR1:GUS* gene was observed on the medium-treated plants (negative control) (Figure 6).

### Effect of JCK-6131 on Defense Responses in Tomato Against *Ralstonia solanacearum*

**FIGURE 7:**
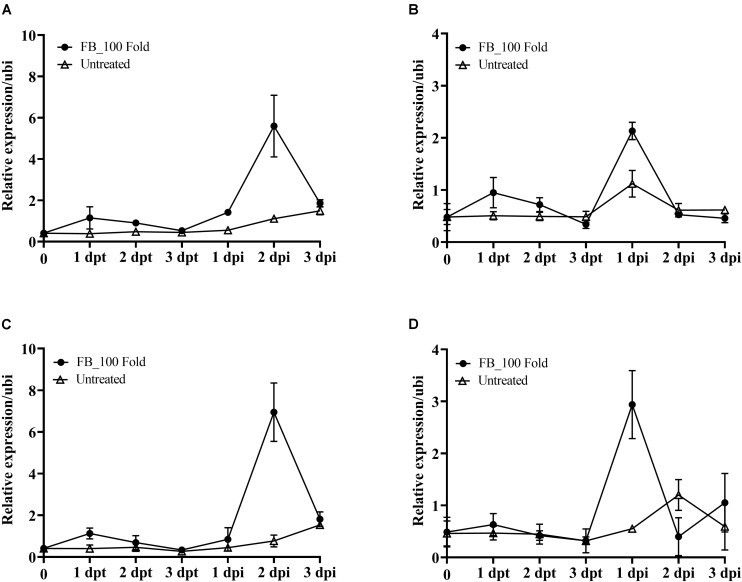
Effect of the fermentation broth of *Streptomyces* sp. JCK-6131 on the gene expression levels of tomato plants before and after inoculation by *Ralstonia solanacearum*. The expression of the *PR1* **(A)**, *PR3* **(B)**, *PR5* **(C)**, and *PR12* **(D)** genes was analyzed from plants sprayed with 1% GSS (untreated) and treatment with 100-fold-diluted (FB_100 Fold) fermentation broth at different times, including 1, 2, and 3 days post treatment (dpt), and 1, 2, and 3 days post inoculation (dpi). The results display the average values ± SD (*n* = 3) of three runs with three replicates.

According to the above results, the fermentation broth of JCK-6131 induced the expression of the *PR1* gene. To clearly elucidate whether JCK-6131 plays a role in enhancing plant defense against pathogens, we analyzed the expression pattern of *PR* genes related to response to JCK-6131 treatment using RT-qPCR. The *PR* genes analyzed were *PR1*, *PR3*, *PR5*, and *PR1*2, which are signature genes of the SA and JA signaling pathways. Tomato plants were foliar-sprayed with the 100-fold-diluted fermentation broth of JCK-6131, and the leaves were collected to measure the transcript levels of *PR* genes at 1, 2, and 3 days post-treatment (dpt) and post-inoculation (dpi). Compared with the negative control, the fermentation broth of JCK-6131 slightly induced the expression of *PR1*, *PR3*, *PR5*, and *PR1*2 genes in tomato plants at 1 dpt; however, the expression levels of these genes gradually decreased at 2 and 3 dpt. The expression levels of *PR1* and *PR5* were induced at higher levels than those of *PR3* and *PR1*2. The expression levels of *PR1* and *PR5* in JCK-6131-treated plants were 2.99 and 2.80 times higher than those in the untreated plants, respectively (Figures 7A,C). The transcription levels of the *PR3* and *PR1*2 genes were 1.88 and 1.35 times higher in the treated plants, respectively, compared to those in the untreated plants (Figures 7B,D).

To elucidate whether pre-treatment with the fermentation broth of JCK-6131 played a role in supporting plant defenses against pathogen infection, we further analyzed the transcription levels of four marker genes following the inoculation with Rs. Increased expression of target genes was observed in both the pretreated and untreated plants as a result of pathogen attack. The upregulation of *PR* genes in JCK-6131-treated plants was faster and stronger than in the untreated plants. In JCK-6131-treated plants, the expression levels of the *PR3* and *PR1*2 genes reached a maximum at 1 dpi and were then downregulated at 2 and 3 dpi (Figures 7B,D); their transcription levels were higher than those in the untreated plants by 1.9 and 5.3 times, respectively. In contrast, the expression levels of *PR1* and *PR5* in the JCK-6131-treated plants reached a maximum at 2 dpi, at which time it was 5.05 and 5.32 times higher, respectively, compared to those in the untreated plants (Figures 7A,C).

## Discussion

Given their abundance, biodiversity, and wide distribution in the natural environment, beneficial bacteria are promising candidates for plant protection (Compant et al., 2005). *Streptomyces* spp. have a great potential in the control of plant bacterial and fungal diseases by producing numerous antibiotics that can kill or inhibit the growth of plant pathogens (Kim et al., 1999; Dhanasekaran et al., 2012; Aæimoviæ et al., 2015). *Streptomyces*-derived antibiotics were also reported to act as weapons in mediating *Streptomyces-Fusarium* interactions in soil (Essarioui et al., 2020).

We discovered a *Streptomyces* strain exhibiting a broad-spectrum antagonistic activity against various phytopathogenic bacteria and fungi. The fermentation broth of JCK-6131 effectively suppressed the development of fire blight of apple, bacterial wilt of tomato, and *Fusarium* wilt of cucumber. Together with that, the PPE also displayed effective disease control efficacy of tomato bacterial wilt. Additionally, the JCK-6131 strain can promote plant growth by producing IAA (data not shown), indicating that it belonged to the PGPB group. Identification of antimicrobial compounds showed that the strain produces streptothricin antibiotics, which have broad-spectrum antimicrobial activity against bacteria and fungi. Three compounds were identified: streptothricin E acid, streptothricin D, and 12-carbamoylstreptothricin D. Till date, many members of the streptothricin group have been identified. Their chemical structures consist of β-lysine, streptolidine, and ?-glucosamine; the number of β-lysine chains is known to play an important role in antibacterial activity (Ji et al., 2009). Moreover, streptothricins were tested as a fungicide in agriculture for controlling plant diseases and registered as an agrochemical in China in the 1990s (Ji et al., 2008).

Apart from their ability to produce antibiotics, *Streptomyces* spp. have also been recognized to induce the expression of defense mechanism-associated genes in plants (Vurukonda et al., 2018). They can directly trigger or produce elicitors that can induce systemic resistance in the plant against pathogens (Dias et al., 2017; Abbasi et al., 2019; Vergnes et al., 2020). Nasr-Eldin et al. (2019) reported that the foliar spraying of the fermentation filtrates of *Streptomyces* spp. showed effective protection against potato-virus Y in all potato cultivars. The disease incidence caused by Rs in tomato plants was reduced after pre-treatment with the cell suspensions of *Streptomyces flavotricini* vh8 and *Streptomyces toxytricini* vh8 (Patil et al., 2011). Similarly, in the present study, the treatment with the 100-fold-diluted JCK-6131 fermentation broth suppressed the development of tomato bacterial wilt and cucumber *Fusarium* wilt by approximately 50%. Additionally, the disease control efficacy of the 100-fold-diluted JCK-6131 fermentation broth against tomato bacterial wilt was similar, regardless of the method of its application (foliar spraying or soil drenching). These results suggest that strain JCK-6131 could enhance defense resistance in plants.

Interactions between plants and pathogens results in the activation of defense signaling pathways such as the SA and JA signaling pathways, which leads to the accumulation of PR proteins that inhibit the growth of pathogens or repress the spread of disease to other organs (González-Bosch, 2018). The expression of *PR* genes is markedly induced by both biotic and abiotic stresses, making them important molecular markers for defense signaling pathways in plants (Van Loon and Van Strien, 1999). Among *PR* genes, the expression of the *PR1* gene has commonly been used as a marker of the activation of SAR, which enhances plant resistance to various pathogens (Park and Kloepper, 2000; Molinari et al., 2014; Li et al., 2015; Ali et al., 2018). In the present study, we found that the fermentation broth of the JCK-6131 strain induced the expression of the *PR1:GUS* gene in the transgenic plants, indicating that the SA signaling pathway was activated. A similar phenomenon was also reported by Vergnes et al. (2020), *PR1:GUS* gene expression in *Arabidopsis thaliana* plants was induced by 1% fermentation broth of *Streptomyces* sp. AgN23. Thus, these results suggest that JCK-6131 is capable of producing SAR elicitors.

The signaling crosstalk between the SA- and JA-signaling pathways are both synergistic and antagonistic. This crosstalk provides the potential for efficient regulation in plant defense responses according to the type of pathogen, and it depends on the relative concentration of these phytohormones (Li et al., 2019). Mur et al. (2006) reported that treatment with a relatively low concentration of both SA and JA resulted in the synergistic expression of the *PR1* and *defensin* genes in tobacco plants, whereas a high concentration of these two phytohormones led to an antagonistic effect. We observed the activation of both the SA and JA signaling pathways, as evident by the expression of their signature genes *PR1*, *PR3*, *PR5*, and *PR1*2. The expression of marker genes for both pathways in this study showed a synergistic effect at a relatively moderate level in the tomato plant. In contrast, the overexpression of marker genes for the SA pathway led to a significant reduction in the expression of JA-marker genes at 2 dpi. Conversely, the fermentation broth of JCK-6131 induced the early and weak expression of the *PR1*, *PR3*, *PR5*, and *PR1*2 genes in tomato plants 1 day after treatment. This suggests that both the SA- and JA-signaling pathways may be activated during priming with JCK-6131.

Van Wees et al. (2008) reported that the activation of both the SA and JA/ET pathways resulted in enhanced protection capability compared with the independent activation of each of these pathways. However, depending on the type of pathogen, the plant defense system will activate the suitable signaling pathway for infection characteristics. Ahn et al. (2011) and Liu et al. (2017) reported that Rs infection resulted in the early simultaneous expression of genes associated with the SA and JA signaling pathways in tomato and tobacco plants, respectively. In the present study, we found that the simultaneous expression of SA and JA marker genes was observed at 1 dpi, while they displayed an antagonistic effect at 2 dpi when the target genes of the SA-signaling pathway were strongly expressed. The expression of *PR* genes in JCK-6131-primed plants was faster and stronger than that of those in the unprimed plants after Rs inoculation. Because defense priming is considered a state of getting ready for battle, it contributes to enhancing plant resistance against a broad spectrum of pathogens. Therefore, primed plants display a more rapid and robust defense than unprimed plants when they are attacked by pathogens (Conrath et al., 2015). In this study, it was confirmed that both the SA and JA signaling pathways are involved in priming with JCK-6131.

## Conclusion

In this study, we show that *Streptomyces* sp. JCK-6131 can effectively protect plants from bacterial and fungal pathogens via two major mechanisms. The first mechanism was the production of streptothricin antibiotics, which possessed a strong and broad-spectrum activity against various phytopathogenic bacteria and fungi. The second mechanism was the induction of plant systemic resistance against bacterial and fungal pathogens. Further studies regarding the identification of active metabolites responsible for plant resistance, optimization of fermentation culture conditions, development of optimum formulation, and evaluation of disease control efficacy of such metabolites against various plant diseases in fields are necessary.

## Data Availability Statement

The original contributions presented in the study are included in the article/Supplementary Material, further inquiries can be directed to the corresponding author/s.

## Author Contributions

KDL and J-CK designed the study and wrote and revised the manuscript. KDL designed and performed experiments and analyzed the data. NHY, HTN, and KDL performed the RNA isolation and identified *Streptomyces* species. CWL and JK did the LC-MS/MS analysis. All authors read and approved the final version of this manuscript.

## Conflict of Interest

The authors declare that the research was conducted in the absence of any commercial or financial relationships that could be construed as a potential conflict of interest. The authors also declare that they will apply for one patent using the results of this study.

## Publisher’s Note

All claims expressed in this article are solely those of the authors and do not necessarily represent those of their affiliated organizations, or those of the publisher, the editors and the reviewers. Any product that may be evaluated in this article, or claim that may be made by its manufacturer, is not guaranteed or endorsed by the publisher.

## Supplementary Material

Supplementary Figure 1 ESI-MS/MS fragmentation pattern of streptothricin E acid.

Supplementary Figure 2 ESI-MS/MS fragmentation pattern of streptothricin D.

Supplementary Figure 3 ESI-MS/MS fragmentation pattern of 12-carbamoylstreptothricin D.

Supplementary Table 1 Primer sequences used in RT-qPCR analysis.

## References

[B1] Abbasi S. Safaie N. Sadeghi A. Shamsbakhsh M. (2019). Streptomyces strains induce resistance to *Fusarium oxysporum f. sp. lycopersici* Race 3 in tomato through different molecular mechanisms. *Front. Microbiol.* 10:1505. 10.3389/fmicb.2019.01505 31333615 PMC6616268

[B2] Abro M. A. Sun X. Li X. Jatoi G. H. Guo L.-D. (2019). Biocontrol potential of fungal endophytes against *Fusarium oxysporum* f. sp. cucumerinum causing wilt in cucumber. *Plant Pathol. J.* 35 598–608. 10.5423/PPJ.OA.05.2019.0129 31832040 PMC6901257

[B3] Aæimoviæ S. G. Zeng Q. McGhee G. C. Sundin G. W. Wise J. C. (2015). Control of fire blight (*Erwinia amylovora*) on apple trees with trunk-injected plant resistance inducers and antibiotics and assessment of induction of pathogenesis-related protein genes. *Front. Plant Sci* 6:16. 10.3389/fpls.2015.00016 25717330 PMC4323746

[B4] Ahn I. P. Lee S. W. Kim M. G. Park S. R. Hwang D. J. Bae S. C. (2011). Priming by rhizobacterium protects tomato plants from biotrophic and necrotrophic pathogen infections through multiple defense mechanisms. *Mol. Cells* 32 7–14. 10.1007/s10059-011-2209-6 21710203 PMC3887660

[B5] Ali S. Ganai B. A. Kamili A. N. Bhat A. A. Mir Z. A. Bhat J. A. (2018). Pathogenesis-related proteins and peptides as promising tools for engineering plants with multiple stress tolerance. *Microbiol. Res.* 212-213 29–37. 10.1016/j.micres.2018.04.008 29853166

[B6] Bruce T. J. A. Pickett J. A. (2007). Plant defence signalling induced by biotic attacks. *Curr. Opin. Plant Biol.* 10 387–392. 10.1016/j.pbi.2007.05.002 17627867

[B7] Cabrefiga J. Montesinos E. (2017). Lysozyme enhances the bactericidal effect of BP100 peptide against *Erwinia amylovora*, the causal agent of fire blight of rosaceous plants. *BMC Microbiol.* 17:39. 10.1186/s12866-017-0957-y 28212623 PMC5316217

[B8] Chaparro J. M. Badri D. V. Vivanco J. M. (2014). Rhizosphere microbiome assemblage is affected by plant development. *ISME J.* 8 790–803. 10.1038/ismej.2013.196 24196324 PMC3960538

[B9] Cheng J. Yang S. H. Palaniyandi S. A. Han J. S. Yoon T.-M. Kim T.-J. (2010). Azalomycin F complex is an antifungal substance produced by Streptomyces malaysiensis MJM1968 isolated from agricultural soil. *J. Kor. Soc. Appl. Biol.Chem.* 53 545–552. 10.3839/jksabc.2010.084

[B10] Compant S. Duffy B. Nowak J. Clément C. Barka E. A. (2005). Use of plant growth-promoting bacteria for biocontrol of plant diseases: Principles, mechanisms of action, and future prospects. *Appl. Environ. Microbiol.* 71:4951. 10.1128/AEM.71.9.4951-4959.2005 16151072 PMC1214602

[B11] Conrath U. Beckers G. J. M. Langenbach C. J. G. Jaskiewicz M. R. (2015). Priming for enhanced defense. *Annu. Rev. Phytopathol.* 53 97–119. 10.1146/annurev-phyto-080614-120132 26070330

[B12] De Vos M. Van Oosten V. R. Van Poecke R. M. Van Pelt J. A. Pozo M. J. Mueller M. J. (2005). Signal signature and transcriptome changes of *Arabidopsis* during pathogen and insect attack. *Mol. Plant Microb. Interact.* 18 923–937. 10.1094/mpmi-18-0923 16167763

[B13] Dhanasekaran D. Thajuddin N. Panneerselvam A. (2012). “Applications of actinobacterial fungicides in agriculture and medicine, in fungicides for plant and animal diseases,” in *Fungicides for Plant and Animal Diseases*, ed. Dharumadurai D. Dr (London: InTech Open), 10.5772/25549

[B14] Dias M. P. Bastos M. S. Xavier V. B. Cassel E. Astarita L. V. Santarém E. R. (2017). Plant growth and resistance promoted by *Streptomyces* spp. in tomato. *Plant Physiol. Biochem.* 118 479–493. 10.1016/j.plaphy.2017.07.017 28756346

[B15] Dong C.-J. Wang L.-L. Li Q. Shang Q.-M. (2019). Bacterial communities in the rhizosphere, phyllosphere and endosphere of tomato plants. *PLoS One* 14:e0223847. 10.1371/journal.pone.0223847 31703074 PMC6839845

[B16] Essarioui A. LeBlanc N. Otto-Hanson L. Schlatter D. C. Kistler H. C. Kinkel L. L. (2020). Inhibitory and nutrient use phenotypes among coexisting *Fusarium* and *Streptomyces* populations suggest local coevolutionary interactions in soil. *Environ. Microbiol.* 22 976–985. 10.1111/1462-2920.14782 31424591

[B17] González-Bosch C. (2018). Priming plant resistance by activation of redox-sensitive genes. *Free Radic. Biol. and Med.* 122 171–180. 10.1016/j.freeradbiomed.2017.12.028 29277443

[B18] Ji Z. Wang M. Wei S. Zhang J. Wu W. (2009). Isolation, structure elucidation and antibacterial activities of streptothricin acids. *J. Antibiot.* 62 233–237. 10.1038/ja.2009.16 19300469

[B19] Ji Z. Wei S. Zhang J. Wu W. Wang M. (2008). Identification of streptothricin class antibiotics in the earlystage of antibiotics screening by electrospray ionization mass spectrometry. *J. Antibiot.* 61 660–667. 10.1038/ja.2008.93 19168980

[B20] Kim B. S. Moon S. S. Hwang B. K. (1999). Isolation, identification, and antifungal activity of a macrolide antibiotic, oligomycin A, produced by *Streptomyces libani*. *Can. J. Bot.* 77 850–858. 10.1139/b99-044 33356898

[B21] Kim J. Le K. D. Yu N. H. Kim J. I. Kim J. C. Lee C. W. (2020). Structure and antifungal activity of pelgipeptins from *Paenibacillus elgii* against phytopathogenic fungi. *Pestic. Biochem. Phys*. 163 154–163. 10.1016/j.pestbp.2019.11.009 31973853

[B22] Kondo Y. Ito T. Nakagami H. Hirakawa Y. Saito M. Tamaki T. (2014). Plant GSK3 proteins regulate xylem cell differentiation downstream of TDIF–TDR signalling. *Nat. Commun.* 5:3504. 10.1038/ncomms4504 24662460

[B23] Lazarovits G. Turnbull A. Johnston-Monje D. (2014). *Plant Health Management: Biological Control of Plant Pathogens.* Oxford: Academic Press.

[B24] Le K. D. Kim J. Yu N. H. Kim B. Lee C. W. Kim J. C. (2020). Biological control of tomato bacterial wilt, Kimchi cabbage soft rot, and red pepper bacterial leaf spot using *Paenibacillus elgii* JCK-5075. *Front. Plant Sci.* 11:775. 10.3389/fpls.2020.00775 32714339 PMC7340725

[B25] Lee I. Ouk Kim Y. Park S.-C. Chun J. (2016). OrthoANI: an improved algorithm and software for calculating average nucleotide identity. *Int. J. Syst. Evol. Microbiol.* 66 1100–1103. 10.1099/ijsem.0.000760 26585518

[B26] Lee J. H. Kim J.-C. Jang K. S. Choi Y. H. Choi G. J. (2014). Efficient screening method for resistance of cucumber cultivars to Fusarium oxysporum f. sp. cucumerinum. *Res. Plant Dis.* 20 245–252. 10.5423/RPD.2014.20.4.245

[B27] Li N. Han X. Feng D. Yuan D. Huang L.-J. (2019). Signaling crosstalk between salicylic acid and ethylene/jasmonate in plant defense: do we understand what they are whispering? *Int. J. Mol. Sci.* 20:671. 10.3390/ijms20030671 30720746 PMC6387439

[B28] Li Y. Gu Y. Li J. Xu M. Wei Q. Wang Y. (2015). Biocontrol agent Bacillus amyloliquefaciens LJ02 induces systemic resistance against cucurbits powdery mildew. *Front. Microbiol.* 6:883. 10.3389/fmicb.2015.00883 26379654 PMC4551870

[B29] Lima S. M. Melo J. G. Militão G. C. Lima G. M. do Carmo A. L. M. Aguiar J. S. (2017). Characterization of the biochemical, physiological, and medicinal properties of *Streptomyces hygroscopicus* ACTMS-9H isolated from the Amazon (Brazil). *Appl. Microbiol. Biotechnol.* 101 711–723. 10.1007/s00253-016-7886-9 27757508

[B30] Liu Q. Liu Y. Tang Y. Chen J. Ding W. (2017). Overexpression of NtWRKY50 increases resistance to *Ralstonia solanacearum* and alters salicylic acid and jasmonic acid production in tobacco. *Front. Plant. Sci.* 8:1710. 10.3389/fpls.2017.01710 29075272 PMC5641554

[B31] Molinari S. Fanelli E. Leonetti P. (2014). Expression of tomato salicylic acid (SA)-responsive pathogenesis-related genes in Mi-1-mediated and SA-induced resistance to root-knot nematodes. *Mol. Plant Pathol.* 15 255–264. 10.1111/mpp.12085 24118790 PMC6638815

[B32] Mur L. A. J. Kenton P. Atzorn R. Miersch O. Wasternack C. (2006). The outcomes of concentration-specific interactions between salicylate and jasmonate signaling include synergy, antagonism, and oxidative stress leading to cell death. *Plant Physiol.* 140 249. 10.1104/pp.105.072348 16377744 PMC1326048

[B33] Mwajita M. R. Murage H. Tani A. Kahangi E. M. (2013). Evaluation of rhizosphere, rhizoplane and phyllosphere bacteria and fungi isolated from rice in Kenya for plant growth promoters. *SpringerPlus* 2:606. 10.1186/2193-1801-2-606 24349944 PMC3863402

[B34] Nasr-Eldin M. Messiha N. Othman B. Megahed A. Elhalag K. (2019). Induction of potato systemic resistance against the potato virus Y (PVYNTN), using crude filtrates of Streptomyces spp. under greenhouse conditions. *Egypt J. Biol. Pest Control* 29:62. 10.1186/s41938-019-0165-1

[B35] Nishad R. Ahmed T. Rahman V. J. Kareem A. (2020). Modulation of plant defense system in response to microbial interactions. *Front. Plant Sci.* 11:1298. 10.3389/fmicb.2020.01298 32719660 PMC7350780

[B36] Niu D.-D. Liu H.-X. Jiang C.-H. Wang Y.-P. Wang Q.-Y. Jin H.-L. (2011). The plant growth–promoting rhizobacterium *Bacillus cereus* AR156 induces systemic resistance in *Arabidopsis thaliana* by simultaneously activating salicylate– and jasmonate/ethylene-dependent signaling pathways. *Mol. Plant Microb. Interact.* 24 533–542. 10.1094/mpmi-09-10-0213 21198361

[B37] Park K. S. Kloepper J. W. (2000). Activation of PR-1a promoter by rhizobacteria that induce systemic resistance in tobacco against *Pseudomonas* syringae pv. tabaci. *Biol. Control* 18 2–9. 10.1006/bcon.2000.0815

[B38] Patil H. J. Srivastava A. K. Singh D. P. Chaudhari B. L. Arora D. K. (2011). Actinomycetes mediated biochemical responses in tomato (*Solanum lycopersicum*) enhances bioprotection against *Rhizoctonia solani*. *Crop Prot.* 30 1269–1273. 10.1016/j.cropro.2011.04.008

[B39] Suárez-Moreno Z. R. Vinchira-Villarraga D. M. Vergara-Morales D. I. Castellanos L. Ramos F. A. Guarnaccia C. (2019). Plant-growth promotion and biocontrol properties of three *Streptomyces* spp. isolates to control bacterial rice pathogens. *Front. Microbiol.* 10:290. 10.3389/fmicb.2019.00290 30858835 PMC6398372

[B40] Ul Haq I. Sarwar M. K. Faraz A. Latif M. Z. (2020). “Synthetic chemicals: major component of plant disease management,” in *Plant Disease Management Strategies for Sustainable Agriculture Through Traditional and Modern Approaches*, eds Ul Haq I. Ijaz S. (Cham: Springer International Publishing), 53–81.

[B41] Van Loon L. C. Van Strien E. A. (1999). The families of pathogenesis-related proteins, their activities, and comparative analysis of PR-1 type proteins. *Physiol. Mol. Plant Pathol.* 55 85–97. 10.1006/pmpp.1999.0213

[B42] Van Wees S. C. Van der Ent S. Pieterse C. M. (2008). Plant immune responses triggered by beneficial microbes. *Curr. Opin. Plant Biol.* 11 443–448. 10.1016/j.pbi.2008.05.005 18585955

[B43] Vergnes S. Gayrard D. Veyssière M. Toulotte J. Martinez Y. Dumont V. (2020). Phyllosphere colonization by a coil *Streptomyces* sp. promotes plant defense responses against fungal infection. *Mol. Plant Microb. Interact.* 33 223–234. 10.1094/mpmi-05-19-0142-r 31544656

[B44] Vurukonda S. Giovanardi D. Stefani E. (2018). Plant growth promoting and biocontrol activity of Streptomyces spp. as endophytes. *Int. J. Mol. Sci.* 19:952. 10.3390/ijms19040952 29565834 PMC5979581

[B45] Walters D. R. Ratsep J. Havis N. D. (2013). Controlling crop diseases using induced resistance: challenges for the future. *J. Exp. Bot.* 64 1263–1280. 10.1093/jxb/ert026 23386685

