# Supplementary material for: Streptomyces sp. JCK-6131 Protects Plants Against Bacterial and Fungal Diseases via Two Mechanisms
Source: Front Plant Sci. 2021 Sep 15;12:726266. doi: 10.3389/fpls.2021.726266 (PMC8479116; doi:10.3389/fpls.2021.726266)
Supplement: Supplementary Figure 1 — ESI-MS/MS fragmentation pattern of streptothricin E acid. [file Data_Sheet_1.docx]

**Supplementary Materials**

**Supplementary Table S1.** Primer sequences used in RT-qPCR analysis

| Gene | Sequences 3’ to 5’ |
| --- | --- |
| PR1 | GCCAAGCTATAACTACGCTACCAAC |
|  | GCAAGAAATGAACCACCATCC |
| PR3 | ATGGCGGAAACTGTCCTAGTGGAA |
|  | ACATGGTCTACCATCAGCTTGCCA |
| PR5 | GAGGTTCATGCCAAACTGGTC |
|  | CCGTCAACCAAAGAAATGTCC |
| PR12 | TCACCAAACTATTGGATTTCAA |
|  | GACTCAATTTTTGACTTCTTAATCC |
| UBI | GGACGGACGTACTCTAGCTGAT |
|  | AGCTTTCGACCTCAAGGGTA |

**Supplementary Table S2.** Biochemical and physiological characteristics of *Streptomyces* sp. JCK-6131

| **Medium** | ISP1 | ISP2 | ISP3 | ISP4 | ISP5 | ISP7 |
| --- | --- | --- | --- | --- | --- | --- |
| Color of substrate mycelium | Gray | Gray | White | White | White | Gray |
| Pigment production | Dark Brown | Dark Brown | Light Brown | None | None | Black |
| **Enzymatic production**  Protease  Amylase  Lipase  Cellulase  Chitinase | +  +  +  +  - | | | | | |
| **Carbon source**  D-fructose  D-galactose  D-Manose  D-Xylose  Lactose  L-Arabinose  Maltose  Mannitol  Raffinose  Rhamnose  Sacarose  Starch  Trehalose | +  +  +  +  +  +  +  +  +  +  +  +  + | | | | | |
| **Nitrogen source**  Agrinine  Cysteine  Phenylalanine  Histidine  L-Methyonine  Lysine  Proline  Serine  Theonine  Valine | +  +  +  +  +  +  +  +  +  + | | | | | |
| **NaCl, %**  0  2  4  6  8 | +++  +++  ++  +  + | | | | | |
| **Temperature, ^o^C**  15  20  30  40  45 | +  ++  +++  ++  + | | | | | |
| **pH**  5  7  8  10 | ++  +++  +++  ++ | | | | | |

-: negative; + positive

**Supplementary Figure S1. ESI-MS/MS fragmentation pattern of streptothricin E acid.**


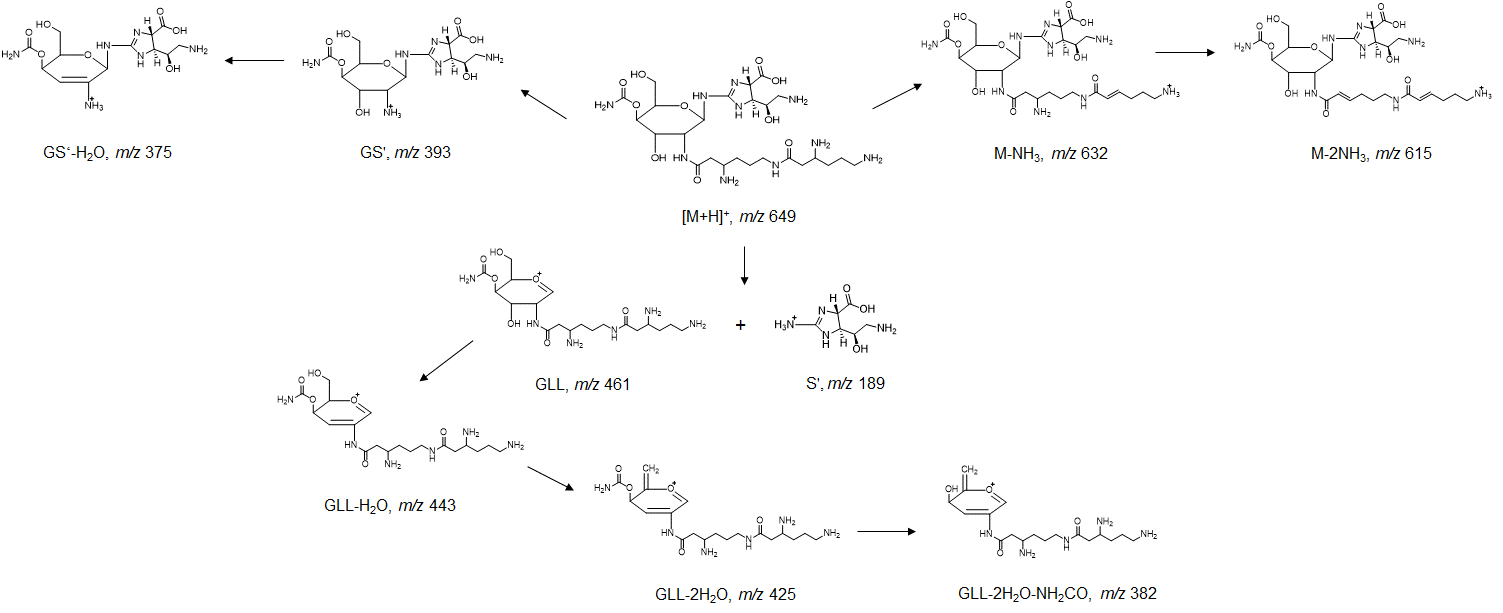


**Supplementary Figure S2. ESI-MS/MS fragmentation pattern of streptothricin D.**


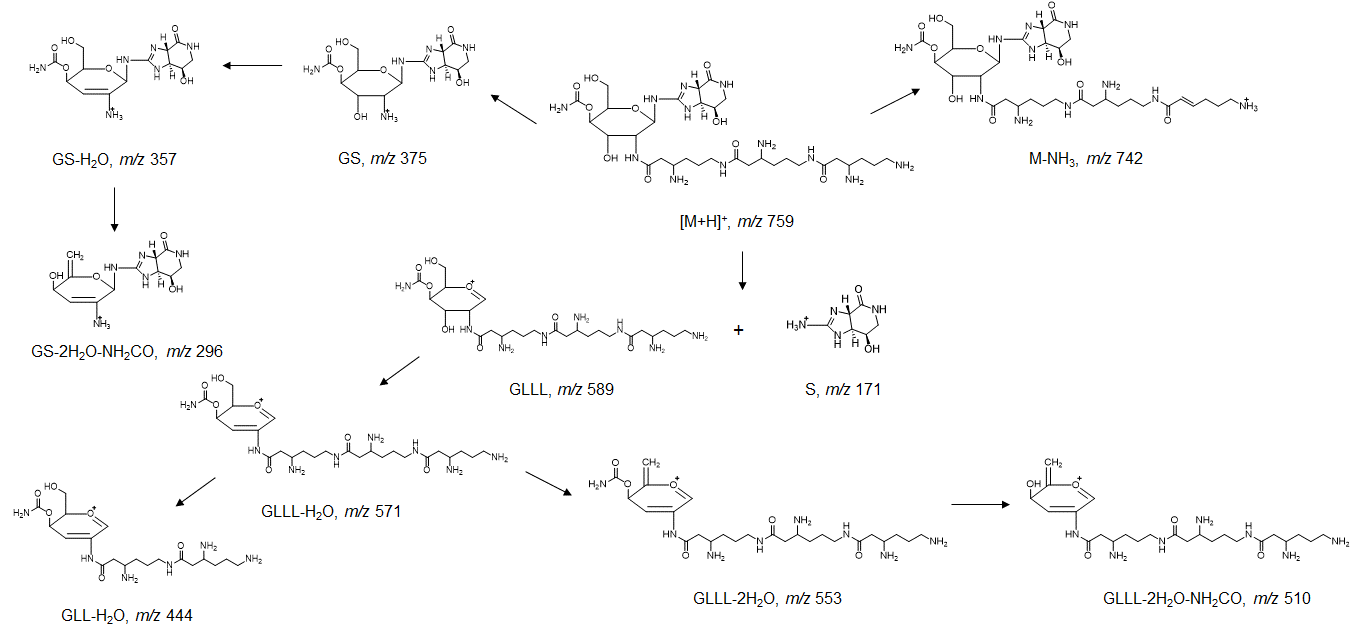


**Supplementary Figure S3. ESI-MS/MS fragmentation pattern of 12-carbamoylstreptothricin D.**


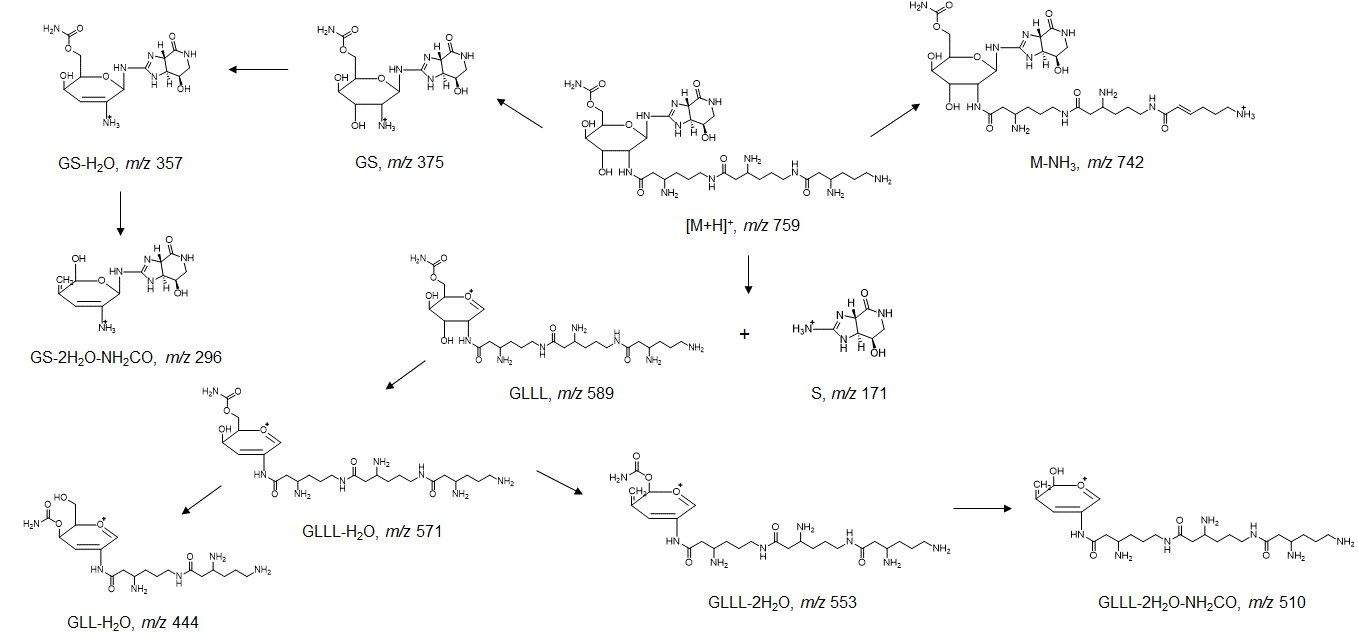


**Supplementary Figure S4.** Control efficacy of the partially purified extract from the fermentation broth of *Streptomyces* sp. JCK-6131 against tomato bacterial wilt disease using soil drenching method. PPE = partially purified extract, Bu = buramycin (× 1000). Values are presented as the mean ± standard error of three runs, with three replicates each. The bars with the same letters represent non-significant differences between the treatments (*p* < 0.05, Fisher’s least significant difference test).
